# False Teeth as Cause of Cancer

**Published:** 1919-03

**Authors:** 


### FALSE TEETH AS CAUSE OF CANCER.

The basis of Field's report is an analysis of a series of 87 clinical cases coming under his observation, in which the initial ulceration or nodule was in close proximity to an artificial denture of which the patient offered complaint. Nineteen showed original development of the tumor adjacent to bridge work and 68 near place work of various types. The malignancy presented in 59 cases in the superin maxilla as against 28 affecting the inferior maxillary region. The tumors were classified as follows: carcinoma, 65; sarcoma, 9; osteoma, 3; fibroma, 4; chondroma 4, and melanosarcoma, 2.—*Journal A. M. A.*, March 1, 1919.

